# Determinants of Sustained Viral Suppression in HIV-Infected Patients with Self-Reported Poor Adherence to Antiretroviral Therapy

**DOI:** 10.1371/journal.pone.0029186

**Published:** 2012-01-03

**Authors:** Tracy R. Glass, Margalida Rotger, Amalio Telenti, Laurent Decosterd, Chantal Csajka, Heiner C. Bucher, Huldrych F. Günthard, Martin Rickenbach, Dunja Nicca, Bernard Hirschel, Enos Bernasconi, Gilles Wandeler, Manuel Battegay, Catia Marzolini, the Swiss HIV Cohort Study

**Affiliations:** 1 Basel Institute for Clinical Epidemiology and Biostatistics, University Hospital of Basel, Basel, Switzerland; 2 Institute of Microbiology, University Hospital of Lausanne, Lausanne, Switzerland; 3 Division of Clinical Pharmacology and Toxicology, University Hospital of Lausanne, Lausanne, Switzerland; 4 Division of Infectious Diseases and Hospital Epidemiology, University of Zurich and University Hospital of Zurich, Zurich, Switzerland; 5 Swiss HIV Cohort Data Center, University Hospital of Lausanne, Lausanne, Switzerland; 6 Division of Infectious Diseases, Kantonsspital of St Gallen, St Gallen, Switzerland; 7 Division of Infectious Diseases, University Hospital of Geneva, Geneva, Switzerland; 8 Division of Infectious Diseases, Regional Hospital of Lugano, Lugano, Switzerland; 9 Division of Infectious Diseases, Bern University Hospital and University of Bern, Bern, Switzerland; 10 Division of Infectious Diseases and Hospital Epidemiology, University Hospital of Basel, Basel, Switzerland; 11 Department of Pharmaceutical Sciences, University of Geneva, Geneva, Switzerland; 12 Institute of Social and Preventive Medicine, University of Bern, Bern, Switzerland; University of Pittsburgh Center for Vaccine Research, United States of America

## Abstract

**Background:**

Good adherence to antiretroviral therapy (ART) is critical for successful HIV treatment. However, some patients remain virologically suppressed despite suboptimal adherence. We hypothesized that this could result from host genetic factors influencing drug levels.

**Methods:**

Eligible individuals were Caucasians treated with efavirenz (EFV) and/or boosted lopinavir (LPV/r) with self-reported poor adherence, defined as missing doses of ART at least weekly for more than 6 months. Participants were genotyped for single nucleotide polymorphisms (SNPs) in candidate genes previously reported to decrease EFV (rs3745274, rs35303484, rs35979566 in *CYP2B6*) and LPV/r clearance (rs4149056 in *SLCO1B1*, rs6945984 in *CYP3A*, rs717620 in *ABCC2*). Viral suppression was defined as having HIV-1 RNA <400 copies/ml throughout the study period.

**Results:**

From January 2003 until May 2009, 37 individuals on EFV (28 suppressed and 9 not suppressed) and 69 on LPV/r (38 suppressed and 31 not suppressed) were eligible. The poor adherence period was a median of 32 weeks with 18.9% of EFV and 20.3% of LPV/r patients reporting missed doses on a daily basis. The tested SNPs were not determinant for viral suppression. Reporting missing >1 dose/week was associated with a lower probability of viral suppression compared to missing 1 dose/week (EFV: odds ratio (OR) 0.11, 95% confidence interval (CI): 0.01–0.99; LPV/r: OR 0.29, 95% CI: 0.09–0.94). In both groups, the probability of remaining suppressed increased with the duration of continuous suppression prior to the poor adherence period (EFV: OR 3.40, 95% CI: 0.62–18.75; LPV/r: OR 5.65, 95% CI: 1.82–17.56).

**Conclusions:**

The investigated genetic variants did not play a significant role in the sustained viral suppression of individuals with suboptimal adherence. Risk of failure decreased with longer duration of viral suppression in this population.

## Introduction

High level of adherence to antiretroviral therapy (ART) is needed to achieve and maintain virological suppression, prevent drug resistance and improve survival in HIV-infected individuals [1]. The minimal level of adherence was initially established as 95% of drug intake and was based on data from non-boosted protease inhibitors (PI) based regimens [1]. More recent studies have indicated that the optimal level of adherence may differ according to the antiretroviral class [2], [3], and should be greater than 95% for non-boosted PI or boosted PI and at least 80% for non-nucleoside reverse transcriptase inhibitors (NNRTI) based regimens [4].

Although nearly perfect adherence to ART is critical for successful HIV treatment, some patients remain suppressed despite poor adherence. The ability to achieve and sustain virological suppression despite suboptimal adherence could result from host genetic and/or pharmacological factors. In fact, the administration of standard doses of most antiretroviral drugs results in a large inter-individual variability in plasma drug concentrations [5], [6]. The reasons for this variability are multifactorial and may involve, besides adherence, factors such as drug-drug interactions, co-morbidities, ethnicity or weight differences, and genetics. In the recent years, genetic polymorphisms in genes coding for drug metabolizing enzymes or transporters have been shown to influence the pharmacokinetics of antiretroviral agents [7]. For instance, a correlation with efavirenz disposition was demonstrated for a 516G>T polymorphism in *CYP2B6* (CYP2B6*6). Individuals with the *CYP2B6* 516TT (homozygous loss of function) genotype had higher efavirenz levels compared to carriers of 516GT (heterozygous loss of function) or 516CC (homozygous reference) genotypes [8]. More recently, the contribution of *CYP2B6* genetic variants to efavirenz disposition was assessed in a pharmacogenetic- pharmacokinetic population analysis, and interestingly, explained half of the inter-individual variability observed with this drug [9]. All together, these factors could explain that some individuals may have higher drug concentrations and may be able to maintain effective levels even after missing doses. Intrinsic drug characteristics such as half-life (long half-life) or drug potency are additional factors that may also contribute to a continuous drug pressure despite suboptimal adherence.

We used the longitudinal nature of the Swiss HIV Cohort Study (SHCS) to select individuals with long periods of self-reported poor adherence to HIV therapy and to investigate the genetic and pharmacological characteristics in those remaining suppressed compared to those not suppressed.

## Methods

### Participants

**Figure 1:**
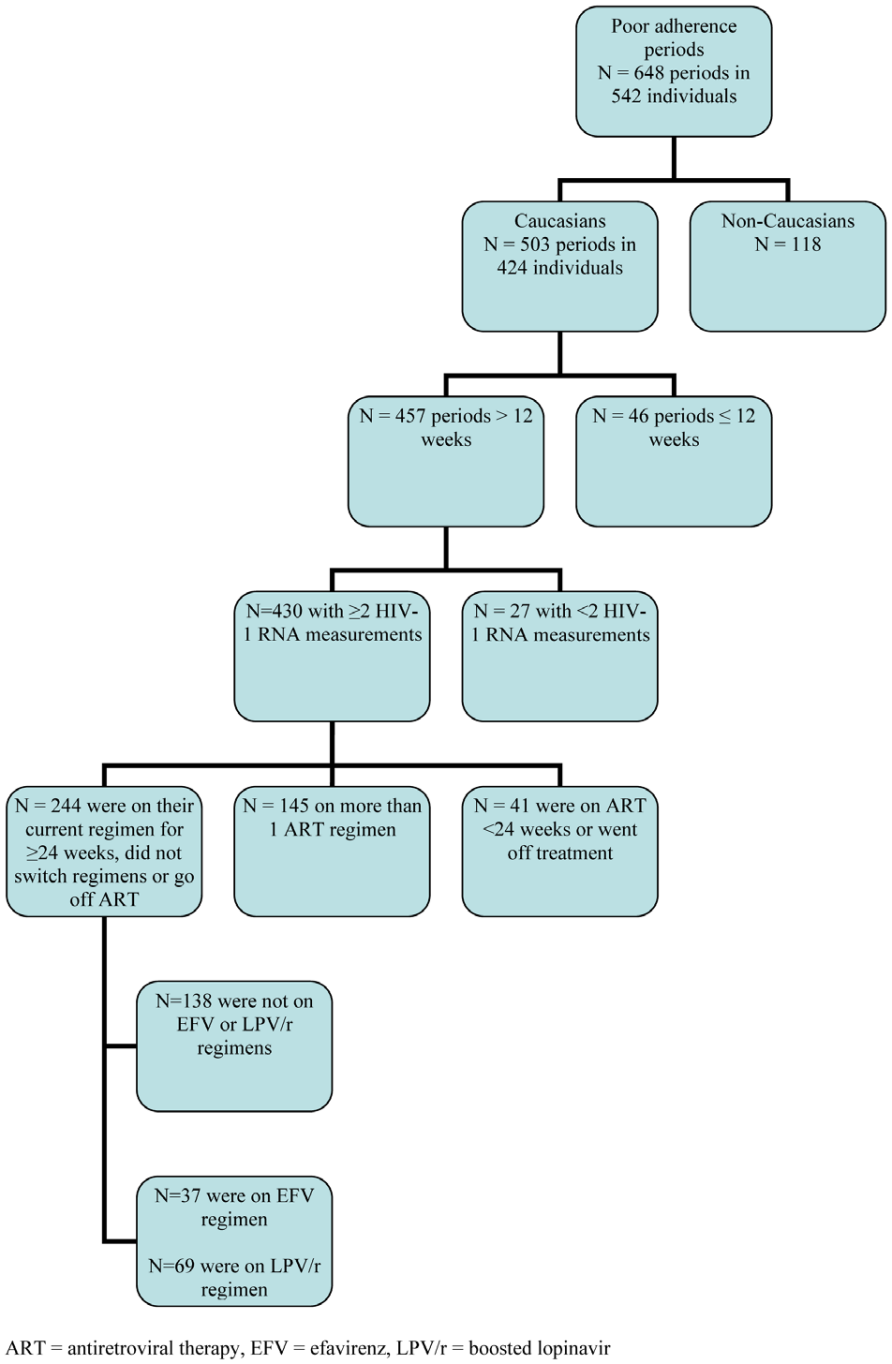
Selection of the study population.

This study included HIV-infected patients enrolled in the SHCS, a nationwide prospective cohort study enrolling HIV-infected individuals aged 18 years or older who are followed-up in HIV clinics (www.SHCS.ch) [10]. Eligible patients were Caucasians, reported poor adherence on at least 2 consecutive follow-up visits more than 3 months apart between January 2003 until May 2009, had at least 2 HIV-1 RNA values during the study period, and were on regimens containing efavirenz (EFV) and/or boosted lopinavir (LPV/r) for more than 24 weeks (see figure 1). The study period was defined for each participant as the first until the last reported date of poor adherence. Participants involved in a drug trial with planned treatment interruptions were not eligible for this study.

### Ethics statement

The ethics committees of all participating centers approved the genetic project and the permission for genetic analyses was approved by the Institutional Review Boards/Ethics committees of the University Hospitals of Basel, Lausanne, Zurich, Bern, Geneva, St Gallen and Lugano. The participants gave written informed consent for genetic testing.

### Genetic analyses

Genotyping of single nucleotide polymorphisms (SNPs) in candidate genes previously reported to decrease EFV (rs3745274, marker of CYP2B6*6; rs35303484, marker of CYP2B6*11 and rs35979566, marker of CYP2B6*15) [9], [11] and LPV/r clearance (rs4149056, marker of SLCO1B1*5; rs6945984 in *CYP3A* and rs717620 in *ABCC2*) [12] was performed using commercially available TaqMan allelic discrimination assays (Applied Biosystems, Foster City, California, USA).

For each drug, the influence of candidate alleles was tested both independently and jointly in a genetic score. Scores for each SNP were defined as 0 = Hom-Ref (homozygous reference) allele, 1 = Het-LOF (heterozygous loss of function) allele or Hom-LOF (homozygous loss of function) allele. For LPV/r, score groups were subsequently classified as: <2 LOF alleles or ≥2 LOF alleles for the combination of the 3 SNPs as done previously [12].

### Drug levels

EFV and LPV/r drug levels were measured during therapeutic drug monitoring only in case of clinical indication and therefore were either not available or regularly documented in all patients. Due to the limited number of drug levels measured within the poor adherence period, we considered all EFV or LPV/r levels available in a given individual. Drug levels are expected to be consistently higher in carriers of genetic variants thus making extrapolation a reasonable approach. Drug levels were quantified by liquid chromatography coupled to tandem mass spectrometry in accordance with previously validated methods [13], [14].

Since plasma levels were measured at random time after last dose intake, observed EFV and LPV/r concentrations were categorized according to percentiles to allow for comparison between individuals. Concentration-time percentile curves (10^th^, 25^th^, 50^th^, 75^th^ and 90^th^ percentiles) were derived using simulations based on previously published population pharmacokinetic models [12], [15] and performed using NONMEM (version VI, Icon Development Solutions, Ellicott City, Maryland, USA). For individuals with multiple drug measurements, the average percentile was calculated.

### Adherence monitoring and definition

Adherence was measured by participants' self-report of non-adherence. During the SHCS follow-up visits, the participants were systematically asked two questions on adherence by their clinicians: (i) How often did you miss a dose of your medication in the last month? Daily, more than once a week, once a week, once every two weeks, once a month, never; and (ii) Did you miss more than one dose in a row? Yes, no. In this study, poor adherence was defined as reporting missing doses of medication either “daily”, “more than once a week”, or “once a week” on at least two consecutive reports. The poor adherence criteria were based on a previous analysis of the SHCS that showed a significantly higher risk of virological rebound in virally suppressed individuals reporting missing doses of ART≥once a week in the last month compared to individuals reporting never missing a dose of ART [16].

### Outcome and covariate definitions

The primary endpoint of the study was virological suppression defined as having all HIV-RNA<400 copies/ml during the study period.

Covariates that were potential confounders of the relationship between host genetic or pharmacological factors and viral suppression were considered for inclusion in the analysis: demographic-related factors (age, gender), patient-related factors (body mass index, current intravenous drug use, co-infection with hepatitis B or C as defined below), condition-related factors (time living with HIV, time on ART, CD4 cell count), and treatment related-factors (prior mono or dual therapy, prior exposure to ART, duration of viral suppression prior to study period, nucleoside backbone, co-medications).

Hepatitis B virus infection was considered active if HBs antigen or HBV DNA were positive. Hepatitis C virus infection was considered active if anti-HCV antibody and HCV RNA were positive and healed if HCV serology was positive and HCV RNA negative. Elevation of liver enzymes (alanine aminostransferase (ALT), aspartate aminotransferase (AST), alkaline phosphatase (ALP)) were graded according to the AIDS adverse event grading table.

### Statistical methods

Logistic regression models were utilized to estimate the effect of host genetic factors and drug levels on viral suppression. Potential confounders of the relationship between host genetic and pharmacological factors were considered for inclusion in the multivariable model although the small sample size limited the number of variables. All analyses were carried out separately for EFV and LPV/r. Estimates of the association between the predictors and outcome are presented with odds ratios (OR) and 95% confidence intervals (CI).

In sensitivity analyses, we also considered an alternative definition for viral suppression - all HIV-1 RNA<50 copies/ml.

All analyses were done with SAS v 9.2 (SAS Institute, Cary, North Carolina) and Stata 10.1 (StataCorp, College Station, Texas).

## Results

### Study population

Over the 6.5 year study period, 648 poor adherence periods from 542 individuals were recorded. The patient selection process is depicted in Figure 1. In the end, 37 individuals on EFV and 69 on LPV/r were included. The median length of poor adherence in both groups was 32 weeks with 18.9% of EFV and 20.3% of LPV/r patients indicating they missed a dose of ART on a daily basis. Demographics of the two populations are described in Table 1.

**Table 1:** Baseline characteristics of the study population.*

Variable	Efavirenz	Lopinavir
N	37	69
Age – median (IQR)	44 (41–54)	42 (38–47)
Male gender - %	83.8	71.0
Risk group for HIV infection- %		
Men having sex with men	40.5	18.8
Heterosexual	46.0	29.0
Injecting drug users	13.5	44.9
Other	0.0	7.3
Past or current IDU - %	16.2	49.3
BMI, kg/m^2^ – mean (SD)	23.2 (4.2)	21.9 (4.1)
Nucleoside backbone - %		
AZT+3TC	54.1	18.8
TDF+3TC	16.2	5.8
TDF+FTC	5.4	5.8
DDI+3TC	5.4	2.9
ABC+TDF	2.7	7.3
ABC+3TC	2.7	4.3
ABC+DDI	0	7.3
DDI+d4T	0	7.3
DDI+TDF	0	7.3
d4T+TDF	0	4.3
Triple NRTI	0	13.0
Other	13.5	15.9
Prior mono or dual therapy - %	32.4	56.5
Prior exposure to NNRTI - %	46.0	21.7
Prior exposure to boosted PI - %	27.0	65.2
Number of previous regimens – median (IQR)	2 (0–4)	4 (1–8)
On co-medication - %	40.5	46.4
Hepatitis C √- %	18.9	52.9
Hepatitis B √ - %	5.6	6.2
Baseline CD4 cell count <200 µ/L - %	8.1	23.2
RNA 400 copies/ml ¶ - %	75.7	55.1
Duration of suppression prior to baseline (months) – median (IQR)	21.2 (9.2–48.1)	13.9 (0–28.4)
Time on ART (years)		
Median (IQR)	4.0 (1.1–7.8)	5.8 (2.5–8.2)
Time living with HIV (years)		
Median (IQR)	8.7 (6.4–12.9)	13.4 (8.7–16.5)

*Baseline is the beginning of the poor adherence period which was defined as 2 consecutive self-reports of missed doses at least 1 per week over at least a 12 week period.

√ See Methods section for definition.

¶ Patients suppressed on the current regimen at baseline and throughout the study period.

IQR = interquartile range, SD = standard deviation, BMI = body max index, IDU = injecting drug use.

3TC = lamivudine, ABC = abacavir, AZT = zidovudine, d4T = stavudine, DDI = didanosine, FTC = emtricitabine, TDF = tenofovir.

In the EFV group, 28/37 (75.7%) maintained a viral load <400 copies/ml throughout the period of poor adherence. In the LPV group, only 38/69 (55.1%) of patients remained suppressed throughout the study period.

### Effect of genetic factors on viral suppression

In the group of patients receiving EFV, genetic variants were found in *CYP2B6* only for rs3745274 with an allele frequency of 25%. In the group of patients receiving LPV/r, genetic variants were found in *SLCO1B1* (rs4149056), *CYP3A* (rs6945984) and *ABCC2* (rs717620) with allele frequencies of 19%, 9% and 22%, respectively (Table 2). Overall, the observed allele frequencies were in good agreement with publicly available SNPs database (www.ncbi.nlm.nih.gov/SNP).

**Table 2:** Adherence, pharmacokinetic, and genetic information.

Variable	Efavirenz	Lopinavir/r
N	37	69
Number of completed adherence questionnaires	2 (2–3)	2 (2–4)
Median (IQR)		
Worst reported missed doses - %		
1 time per week	43.2	29.0
>1 time per week	37.8	50.7
Daily	18.9	20.3
Missed >1 dose in a row in past 4 weeks - %	62.6	56.5
Length of poor adherence period (weeks)		
Median (IQR)	32.0 (27.7–61.0)	32.1 (22.1–58.6)
Drug level √ - percentile value		
Median (IQR)	34.3 (22.5–60.0)	53.8 (32.5–70.0)
Genetic polymorphism †- %		
CYP2B6*6 (rs3745274)		
Hom-Ref	53.1	NA
Het-LOF	43.8	NA
Hom-LOF	3.1	NA
SLC01B1*5 (rs4149056)		
Hom-Ref	NA	64.1
Het-LOF	NA	34.4
Hom-LOF	NA	1.6
*CYP3A* (rs6945984)		
Hom-Ref	NA	81.2
Het-LOF	NA	18.8
Hom-LOF	NA	0
*ABCC2* (rs717620)		
Hom-Ref	NA	59.4
Het-LOF	NA	37.5
Hom-LOF	NA	3.1

√ Average percentile values were used when multiple drug measurements were performed in a single patient. Drug levels available in 30% of participants on EFV and 50% of those on LPV/r treatment.

† EFV: Genetic results available in 86% of patients for the allele CYP2B6*6; No study participants carried a loss/diminished-function CYP2B6*11 or *15 alleles; LPV: Genetic results available in 93% of patients.

IQR = interquartile range, Het = heterozygous, Hom = homozygous, LOF = loss of function, Ref = reference allele. NA = not applicable.

Univariable and multivariable logistic regression analyses for EFV failed to detect an association between 516G>T polymorphism in *CYP2B6* and the likelihood of remaining suppressed (Table 3).

**Table 3:** Logistic regression models for sustained viral suppression (RNA<400 copies/ml) during poor adherence period for those on EFV.

Variable	UnivariableOR (95% CI)	MultivariableOR (95% CI)	Mult.p-value
*CYP2B6* †	1.67 (0.32–8.59)	3.25 (0.10–106.82)	0.51
Low adherence ¶	0.11 (0.01–0.99)	0.11 (0.01–2.03)	0.14
Prior suppression on current ART	3.40 (0.62–18.75)	4.07 (0.36–46.26)	0.26
Prior exposure to NNRTI regimen	0.60 (0.13–2.72)	0.59 (0.04–7.65)	0.68
Length of poor adherence period (weeks)	1.01 (0.98–1.04)	1.03 (0.94–1.13)	0.57
Time since HIV diagnosis (per 5 years)	0.58 (0.29–1.19)	0.38 (0.09–1.61)	0.19

† Reference group for analyses is those with reference allele. Genetic results available in 86% of patients for the allele CYP2B6*6; No study participants carried a loss/diminished-function CYP2B6*11 or CYP2B6*15 alleles.

¶ Low adherence is defined as missing doses of ART more than 1 time per week. Reference group is those who missed a dose of ART 1 time per week.

Univariable and multivariable logistic regression analyses for LPV/r failed to detect an association between each independent SNP and viral suppression (p>0.15) with the exception of the univariable result for SLCO1B1*5 (OR 0.37, 95% CI: 0.13–1.06, p = 0.06). When analyzing the joint effect of the 3 SNPs in a genetic score, the univariable logistic regression showed a trend between a decreased likelihood of viral suppression in carriers of genetic variations (p = 0.08) however the trend did not remain in the multivariable analysis.

### Effect of drug levels on viral suppression

Drug concentrations were only available in 30% of EFV and 50% of LPV/r patients. For most patients, the intra-individual variability in drug levels was very pronounced further supporting the poor adherence pattern in this selected population. No association were found between higher drug levels (expressed as average percentile value) and presence of genetic variants in EFV patients (p = 0.36) or combined genetic variants in LPV/r patients (p = 0.26). The analysis of independent genetic variants in LPV/r patients showed a correlation between carriers of SLCO1B1*5 and higher drug levels (average percentile value in carriers *vs* non carriers: 62 *vs* 42, p = 0.03) but not for carriers of variants in *CYP3A* (p = 0.63) and *ABCC2* (p = 0.61).

No association was found between higher drug levels and the likelihood of remaining suppressed in EFV (p = 0.54) and LPV/r patients (p = 0.12). However, single or repetitive exposure to low drug levels (i.e. ≤10^th^ percentile) was associated with a lower likelihood of viral suppression in LPV/r patients (OR 4.40, 95% CI: 1.04–18.60, p = 0.04). Due to the small number of patients with available drug concentrations, drug levels were not included in the multivariable analyses.

### Effect of adherence on viral suppression

Despite all patients having reported poor adherence, differences in the likelihood of viral suppression could still be seen between the adherence levels (Tables 3 and 4). Individuals on LPV/r missing >1 dose per week were significantly less likely to experience viral suppression than those missing 1 dose a week (OR 0.06, 95% CI: 0.01–0.55, p = 0.01). For EFV patients, a similar trend was found but this was not statistically significant (OR 0.11, 95% CI: 0.01–2.03, p = 0.14).

**Table 4:** Logistic regression models for sustained viral suppression (RNA<400 copies/ml) during poor adherence period for those on LPV.

Variable	UnivariableOR (95% CI)	MultivariableOR (95% CI)	Mult.p-value
Combined variants: ≥2 LOF alleles †	0.38 (0.13–1.11)	0.59 (0.06–5.52)	0.64
Low adherence ¶	0.29 (0.09–0.94)	0.06 (0.01–0.55)	0.01
Prior suppression on current ART	5.65 (1.82–17.56)	8.74 (1.03–74.18)	0.05
Prior exposure to boosted PI	0.48 (0.17–1.34)	0.16 (0.01–1.79)	0.14
Length of poor adherence period (weeks)	1.00 (0.98–1.02)	1.02 (0.99–1.05)	0.26
Age (years)	1.03 (0.98–1.09)	1.20 (1.01–1.41)	0.04
Past or current IDU	0.37 (0.14–0.98)	0.52 (0.09–3.20)	0.48
Taking co medication○	0.33 (0.12–0.88)	0.55 (0.04–7.36)	0.65
CD4 cell count ≥200 µ/L	3.63 (1.10–11.98)	1.75 (0.16–19.38)	0.65
Mild elevation of ALP	0.29 (0.10–0.87)	0.22 (0.03–1.69)	0.15
Time since HIV diagnosis (per 5 years)	0.49 (0.29–0.83)	0.47 (0.17–1.29)	0.14

† Reference group for analyses is those with reference allele or one variant for the combination of the 3 genetic variants. Genetic results available in 93% of patients.

¶ Low adherence is defined as missing doses of ART more than 1 time per week. Reference group is those who missed a dose of ART 1 time per week.

○ Taking any co medication for longer than 30 days during the study period.

ALP = alkaline phosphatase, IDU = injecting drug use, LOF = loss of function.

### Predictors of virological suppression

In univariable models of EFV patients, only low adherence was significantly associated with decreased odds of viral suppression (p = 0.05) (Table 3). However, in multivariable models, the effect was attenuated and was no longer significant (p = 0.14).

In univariable models of LPV/r patients, having low adherence levels, past or current IDU, co-medication, mild elevation of ALP (>1.25–2.5 above the upper limit of normal), or longer time since HIV diagnosis was significantly correlated with a decreased likelihood of remaining suppressed during the period of poor adherence. Prior suppression on the current regimen and higher baseline CD4 cell count were associated with an increased likelihood of remaining suppressed despite poor adherence (Table 4). In multivariable models, only low adherence and prior suppression remained significant whereas older age was associated with an increased likelihood of remaining suppressed.

These findings remained consistent when defining viral suppression as RNA<50 copies/ml.

## Discussion

Nearly perfect adherence to antiretroviral therapy is widely accepted as the primary determinant for achieving and maintaining viral response. However, less is known about the determinants of virological suppression observed paradoxically in some individuals with long periods of self-reported poor adherence to ART. We hypothesized that individuals carrying genetic variants associated with a slow metabolizing profile would maintain sufficient viral inhibitory drug levels even after missing doses.

In our study population, we failed to show a correlation between 516G>T polymorphism in *CYP2B6* and the likelihood of remaining suppressed in patients with self-reported suboptimal adherence to EFV based regimens. The population pharmacokinetic estimates of EFV half-lives have been reported to be 23, 27 and 48 hours for individuals with the *CYP2B6* 516 GG, GT and TT genotypes, respectively [17]. Our study population was mainly characterized by carriers of the *CYP2B6* 516 GG (53%) and 516 GT genotypes (44%) whereas only one patient had the 516 TT genotype (Table 2). Thus, our results suggest that the modest increase in EFV half-life (17%) associated with the *CYP2B6* 516 GT genotype might not be strong enough to compensate for inconsistent adherence by maintaining effective levels until the subsequent dose.

In the multivariable analyses, no correlation was found between independent or combined genetic variants and the likelihood of remaining suppressed in patients with suboptimal adherence to LPV/r based regimens. Although there was a trend to increased drug levels with the SLCO1B1*5 allele, this did not translate in protection from virological failure. One should note that while the genetic determinants of EFV blood levels are well understood [9]), the contribution of genetics to the inter-individual variability of LPV/r pharmacokinetics is modest (less than 5%) [12].

In our population, the likelihood of remaining suppressed was rather related to the degree of adherence. The estimated minimal level of adherence to maintain viral suppression was 86% for EFV and 93% for LPV/r (mainly administered twice daily in our population) as indicated by the increased risk of viral failure in patients missing more than 1 dose a week compared to those missing 1 dose a week (Tables 3 and 4). The lower adherence threshold required for EFV compared to LPV/r was supported by the difference in observed rate of viral suppression in that 76% of the patients receiving EFV versus 55% treated with LPV/r remained suppressed throughout the study period. Our results are consistent with previous studies showing that viral suppression can be reached in some but not all patients at a level of adherence averaging 80% for NNRTI-based regimens [3], [4], [18], [19] and >90% for PI-based regimens [2], [3], [4], [18], [20]. The higher adherence threshold observed in our study might reflect the fact that we used self-reported adherence, which overestimates adherence by 10–20% compared to electronic monitoring [21], [22]. In our study, the degree of adherence rather than consecutive missed doses was a stronger predictor for viral suppression in EFV and LPV/r models. EFV forgiveness of non-adherence has been reported to be likely attributable to its extended half-life [3], [4], [19], whereas LPV/r has a high genetic barrier to resistance which may enhance its forgiveness [23], [24].

Besides the degree of adherence, we found that the likelihood of remaining suppressed was also dependent on the dosing schedule for LPV/r. In our population, LPV/r was administered twice daily (BID) in 64 patients and once daily (QD) in 5 patients. Interestingly, the failure rate was higher in those on a QD regimen with 80% (4/5) of the patients on the LPV/r QD regimen compared to 42% (27/64) on the LPV/r BID regimen not maintaining viral suppression. This observation is compatible with the findings of a recent study comparing the pharmacokinetics of LPV/r BID and QD following drug cessation [25]. Although no participants receiving LPV/r BID had drug levels below the minimal effective concentration (MEC) at 12 hours, 44% of participants receiving LPV/r QD were below the MEC at 24 hours. The higher occurrence of virological failure observed in some clinical trials with the LPV/r QD regimen seems to be related to the fast decay of LPV/r and the achievement of sub-therapeutic concentrations [25].

Interestingly, our data showed that the probability of remaining suppressed increased with the duration of continuous suppression prior to the poor adherence period in both groups, which is in agreement with previous data [26], [27]. It has been postulated that initial treatment during high viral burden likely required higher levels of adherence for full viral suppression than later in chronic treatment when viral burden was less [26], [27]. Furthermore, it has been reported that the latent reservoir and its ability to reactivate upon stimulation with cognate antigen decreases with duration of treatment [28]. Thus, individuals may be more vulnerable to suboptimal adherence shortly after achieving viral suppression but may be able to tolerate missed doses after long-term viral suppression. It should be noted that the probability of remaining suppressed might also be related to inter-individual differences in immune response to HIV virus. Factors such as thymus output, co-infections, residual viral production, age, gender, genetics of immune system, nadir of CD4 cell are known to impact the immune response to HIV [29]. This assumption is supported by the observation that in the univariable model of LPV/r patients, higher baseline CD4 cell count was associated with an increased likelihood of remaining suppressed.

### Limitations

Some limitations of our study should be acknowledged. The stringent selection criteria of our study population (severe non adherence and being steadily on EFV and/or LPV/r) gave us a small sample size and limited power to explore associations in our data. In addition, the limited number of patients with documented drug levels did not allow satisfactory exploration of the role of drug concentration as a predictor of viral suppression. Although, due to the high level of non-adherence, this relationship may have been difficult to quantify precisely.

### Conclusions

The investigated genetic variants did not play a significant role in the sustained viral suppression of individuals with poor adherence. The risk of virological failure decreased with longer duration of viral suppression in this selected population. Patients varied considerably in their prior ART exposure and, although we attempted to adjust for this and the possibility of resistance, we cannot exclude the potential impact of these factors.
